# Case Report - Severe hypospadias in 46, XY karyotype patients: is third level genetic testing always mandatory?

**DOI:** 10.3389/fsurg.2025.1524953

**Published:** 2025-01-31

**Authors:** Giorgia Romano, Giovanni Rollo, Lorna Spagnol, Mafalda Mucciolo, Ottavio Adorisio, Massimiliano Silveri

**Affiliations:** ^1^ Surgical Andrology and Gynecology, Bambino Gesù Children’s Hospital, IRCCS, Rome, Italy; ^2^ Translational Cytogenetics Research Unit, Bambino Gesù Children's Hospital, IRCCS, Rome, Italy

**Keywords:** DSD 46, XY, hypospadias, severe hypospadias, surgery, pediatric, genetic testing

## Abstract

We report a case of a 4-month-old infant with severe genital malformation and a 46, XY karyotype. Genetic testing revealed a variant in the NR5A1 gene, guiding a successful multistage surgical intervention. This case underscores the value of targeted genetic testing in guiding the management of severe hypospadias cases. While genetic investigation isn't routine for all severe hypospadias cases, Next Generation Sequencing (NGS) technologies have influenced the rate of correct diagnoses, reduced diagnostic delay, and helped to determine the need for focused medical care and timely treatment. Too commonly, surgeons tend to attach importance to malformation repair and disregard the genetic diagnoses, but we believe that precise genetic diagnosis improves the accuracy of DSD management in terms of prognostic predictions, the development of an individualized management plan and the determination of treatment options.

## Introduction

The term “disorders/differences of sex development” (DSD) indicates a group of congenital conditions with atypical development of chromosomal, gonadal, or anatomical sex. The 46, XY DSD are characterized by ambiguous external genitalia with a 46, XY karyotype (1). Advances in genomics, particularly NGS, have helped identify multiple genes associated with 46, XY DSD, including NR5A1. Steroidogenic factor 1 (SF-1), encoded by the NR5A1 gene, is a transcription factor crucial for adrenal and gonadal organogenesis (2, 3). NR5A1 mutations have been detected in about 10%–20% of 46, XY DSD cases as the major causes of gonadal dysgenesis with a wide range of clinical phenotypes (4, 5). We present the case of a patient with ambiguous genitalia at birth who was investigated for DSD genetic panel conditions, emphasizing the importance of genetic analysis in understanding and managing DSD cases. This individualized approach is crucial for ensuring the well-being of patients and their families, both physically and mentally (6).

## Case description

### Patient information

A 4-month-old male was referred to our Institution with severe genital malformation.

### Clinical findings

**Figure 1:**
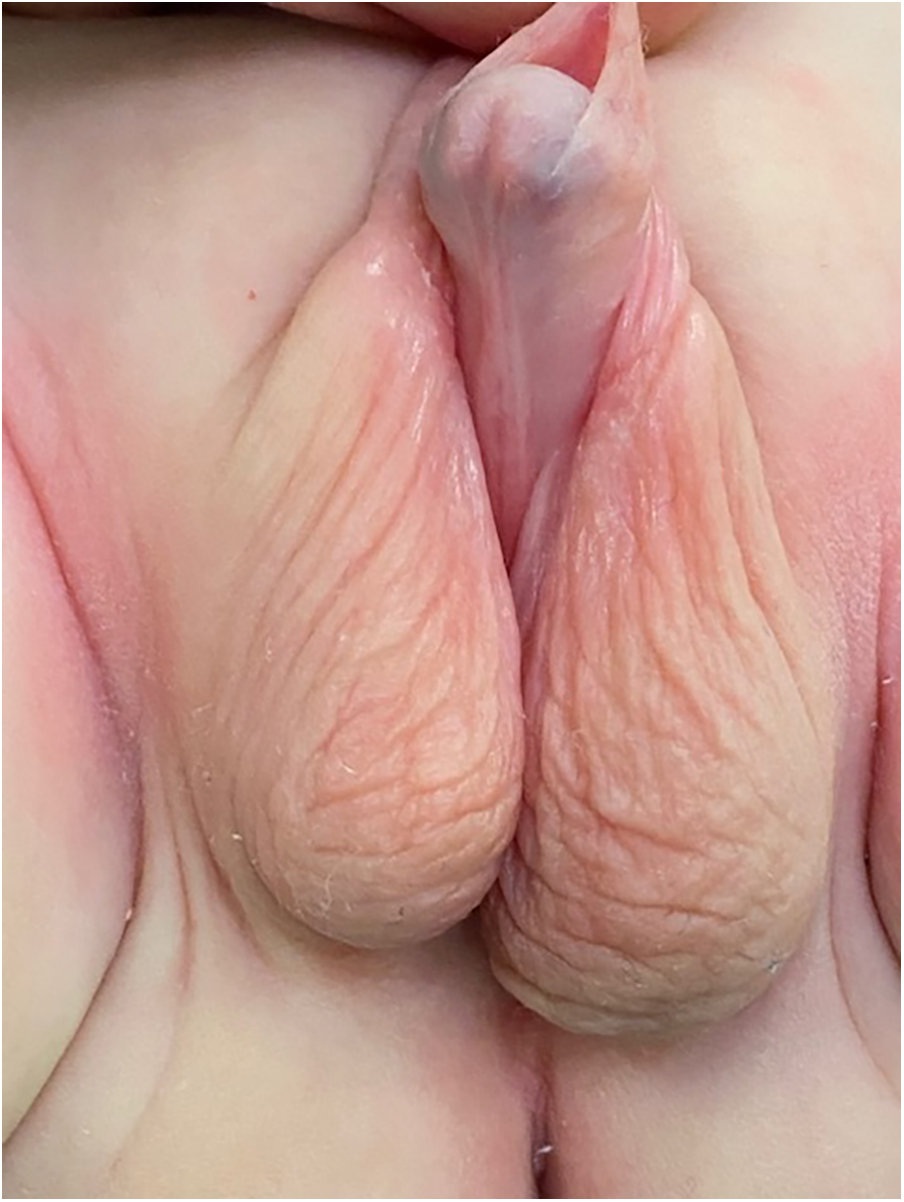
Pre-operative appearance of the external genitalia, complete penoscrotal trasposition.

Genitalia examination revealed a micropenis with complete bifid scrotum and perineal hypospadias (Figure 1). The Glans-Urethral Meatus-Shaft (GMS) hypospadias score (7) was G2M4S4 with complete penoscrotal transposition. Both gonads were palpable at the emergency of the labioscrotal fold and 49 stretched penile length was 2.6 cm.

### Diagnostic approach

The cytogenetics results revealed that the patient had a 46, XY karyotype and laboratory workup showed normal hormone levels. Testicular and scrotal ultrasound examination showed both testes in the scrotal sac, symmetrical and equal in size. We collected blood samples from our patient and analyzed his DNA using a DSD 19-gene panel, NGS identified a heterozygous missense variant in NR5A1 gene, classified as a variant of uncertain significance (VUS).

#### Surgical procedure

**Figure 2:**
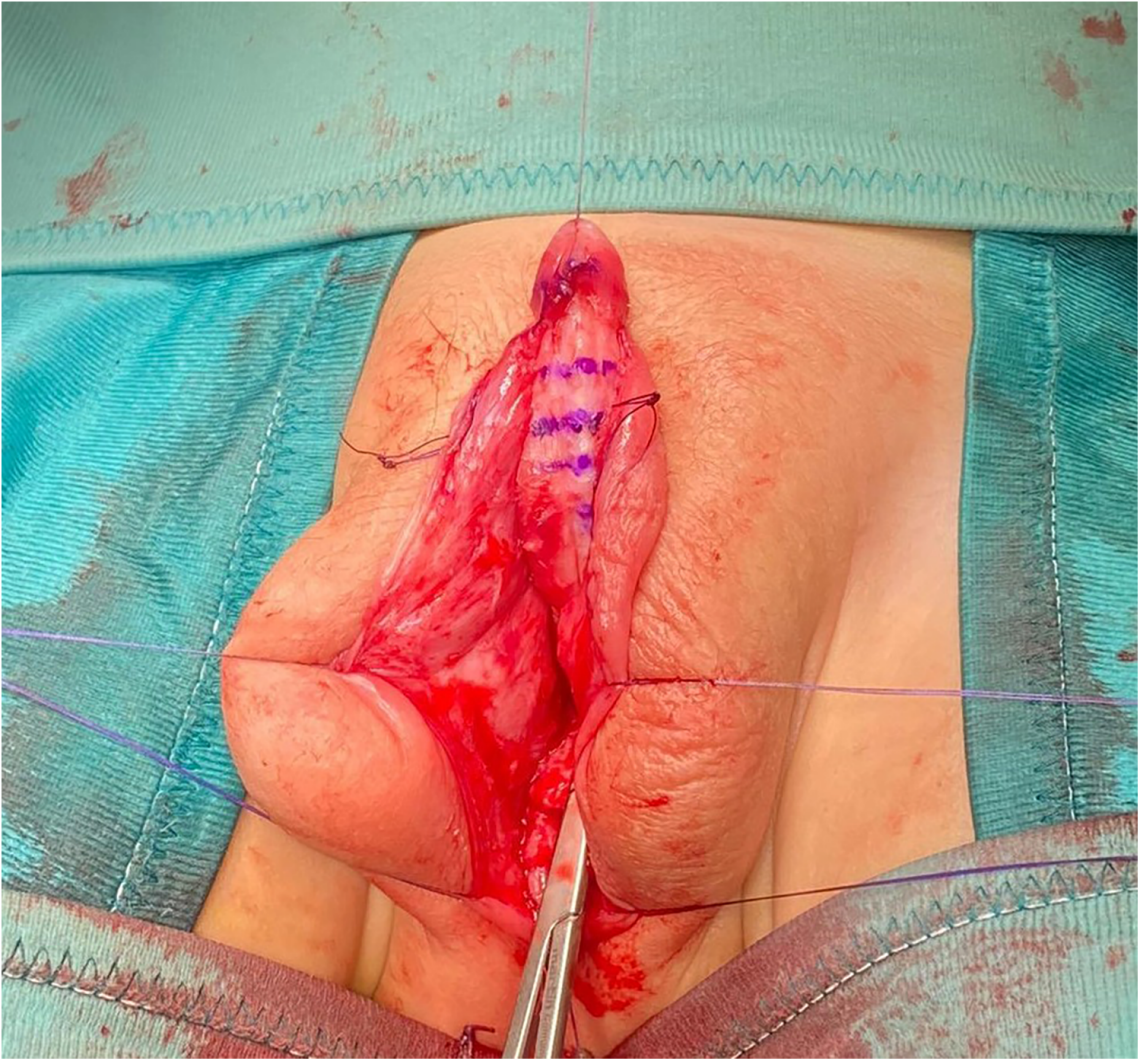
Intra-operative view after the ventral corporotomies.

Multidisciplinary counseling led to surgical intervention, involving a three-stage hypospadias repair (8). In the first stage, at 11 months, a perimeatal incision followed by complete degloving of penile skin up to the level of the penoscrotal junction was made. Artificial erection showed a curvature greater than 60° that was corrected through multiple transverse incisions (corporotomies) of the tunica albuginea in the penile ventral aspect, thus obtaining a 1.2 cm lengthening of the shaft (Figure 2). The corporotomies were covered by a dartos flap from the adjacent shaft skin on one side. Subsequently, a prepuctioplasty was performed: the ventral skin was lengthened with two incisions, the penoscrotal junction was fixed, and the foreskin edges were approximated in two layers ventrally. In the second stage, performed six months after the first step, a dorsal inner preputial flap of 3 × 1.5 cm was harvested and placed ventrally between the proximal meatus and tip of the glans. In the last surgical step, six months after the second surgical step, a U-shaped incision demarcating the neourethra and extending from the original meatus to the distal glans was accomplished. According to the Duplay technique, a three-layer urethral closure was performed. Glanuloplasty and skin cover completed the repair by leaving a wide meatus at the tip of the glans. The urinary diversion was maintained by a urethral catheter and suprapubic cystostomy for 14 days.

### Postoperative outcome

**Figure 3:**
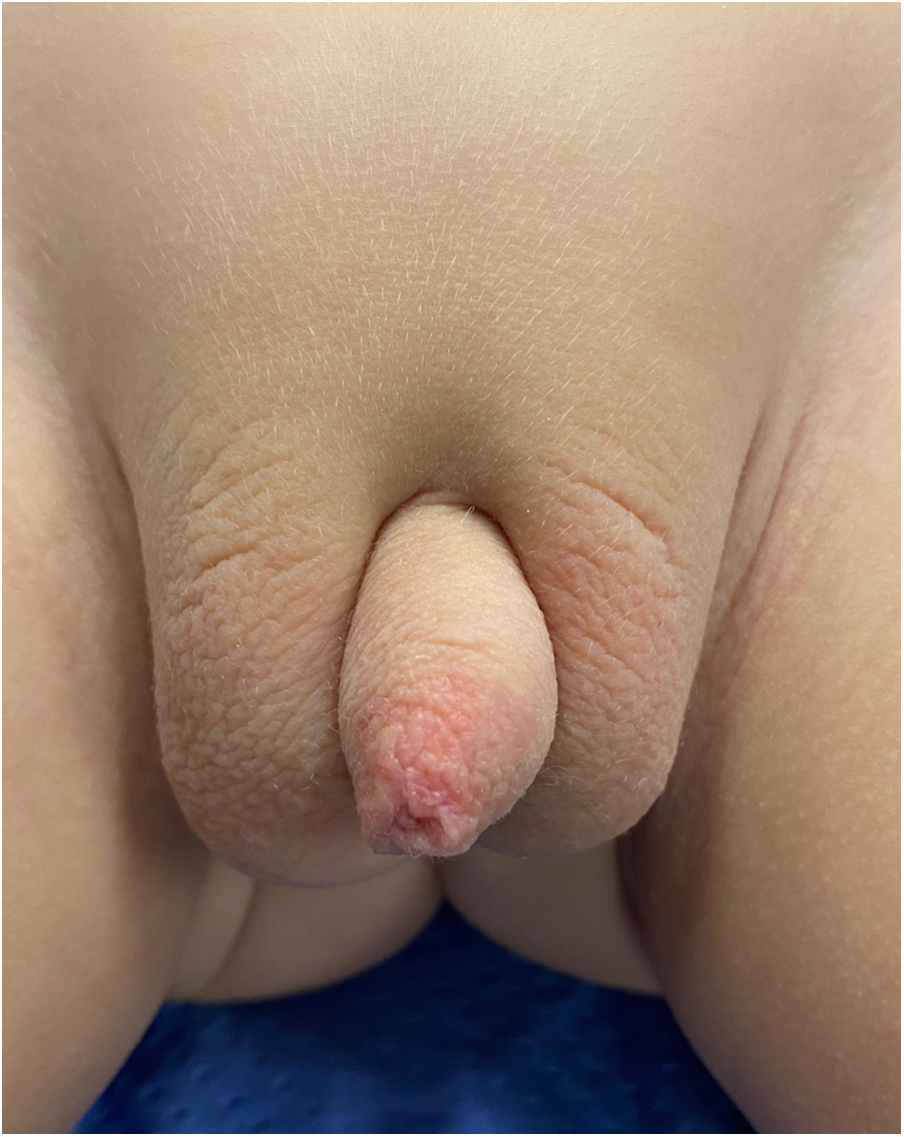
Post-operative appearance after 18-months follow-up.

An 18-month follow-up revealed an uneventful recovery, with a satisfactory genital appearance (Figure 3).

## Discussion

DSDs include a spectrum of phenotypes defined by congenital conditions in which chromosomal, gonadal, or anatomical sex is atypical. Hypospadias is the most frequent form of DSD due to a disruption of the development of the ventral face of the penis in which, the most evident feature is the urethral meatus in an ectopic position. Most often, hypospadias is relatively mild and a genetic cause is not searched or identified (9). However, severe forms, such as posterior and perineal hypospadias with micropenis and significant chordee, represent some of the most severe 46,XY DSDs and may result in ambiguous genitalia at birth. DSDs with a genital appearance that is sufficiently atypical to prompt evaluation occur in approximately 1 in 1,000–4,500 live births (10). The clinical and psychosocial impact of hypospadias is significant, requiring surgical correction typically around one year of age. In severe cases, hypospadias may compromise sexual function, affect quality of life, and impair psychosocial development (11). While most cases are isolated, some are associated with a more complex genetic syndrome involving anomalies of genital development. Moreover, hypospadias may present alongside other abnormalities such as undescended testis, bifid scrotum or inguinal hernia. Overall, less than 10% of cases may be related to genetic defects (12). Familial clustering is present in about 10% of patients with hypospadias. The inheritability is estimated between 57% and 77% of affected patients. Severe hypospadias are generally seen as isolated cases, while milder variants are more often familial (13). The etiology of hypospadias is multifactorial (14, 15). Several genes have been founded to link with hypospadias such as DGKK, ESRs, HOX, ATF3 and VAMP. The precise role of androgen and estrogen signaling that interact in a temporal and tissue-specific manner during genital tubercle development may explain the wide range of meatal/urethral defects. Significant alterations in DNA methylation of sex hormone receptor genes (ESR1 and AR) and fibroblast growth factor (FGFR2 FGF8) may correlate with abnormal expression of these genes in patients with hypospadias. These findings suggest a potential role for epigenetic modifications in hypospadias etiology that, potentially, can be transmitted to future generations. In our case, the patient presented with severe genital malformation, characterized by perineal hypospadias, micropenis, bifid scrotum, and penoscrotal transposition, linked to a heterozygous missense variant in the NR5A1 gene. Although the variant was classified as a variant of uncertain significance (VUS), its identification necessitated a multidisciplinary approach that guided surgical interventions and emphasized the need for long-term follow-up. Mutations in NR5A1 are associated with a broad spectrum of phenotypes, including testicular dysgenesis, progressive endocrine dysfunction and reproductive challenges (3). These factors may also contribute to the risk of gender dysphoria in later life, particularly in cases of severe genital ambiguity. The interplay between physical characteristics, hormonal influences, and psychosocial factors requires ongoing monitoring and psychosocial support. Moreover, the development of gender identity in individuals with NR5A1 mutations may progress over time, necessitating adaptability in management approaches. Comprehensive follow up, including endocrine, psychological, and surgical assessments, is crucial to ensure holistic care and address any emerging concerns, such as declining testosterone production, fertility preservation, or the risk of germ cell tumors (3). Genetic counseling remains essential for the patient and their family, as NR5A1 variants can have implications for female relatives, including the potential for primary ovarian insufficiency (16). The limitations of the studies trying to correlate hypospadias and genetics are that the etiology of hypospadias is obviously multifactorial. These studies are often conducted on a limited number of cases and on a single ethnic group or race. In addition, the various cases of hypospadias are not the same, and the results of the studies are often discordant. To overcome these problems, multicenter studies are needed.

## Conclusion

This case underscores the significance of genetic testing in severe hypospadias, highlighting its contribution to multidisciplinary management and long-term monitoring. The identification of an NR5A1 variant, classified as a variant of uncertain significance (VUS), informed surgical decisions and heightened awareness of possible endocrine complications.

Comprehensive care must emphasize endocrine monitoring, psychosocial support, and fertility preservation. Genetic counseling is essential for addressing familial implications and customizing patient-specific strategies. Additional research is required to delineate more precise correlations between genetic findings and clinical outcomes in severe DSD cases.

## Data Availability

The original contributions presented in the study are included in the article/Supplementary Material, further inquiries can be directed to the corresponding author.
